# Comparative studies on the multi-component pharmacokinetics of *Aristolochiae Fructus* and honey-fried *Aristolochiae Fructus* extracts after oral administration in rats

**DOI:** 10.1186/s12906-017-1626-2

**Published:** 2017-02-10

**Authors:** Jinbin Yuan, Gang Ren, Jian Liang, Chong-Zhi Wang, Zhihong Yan, Qun Huang, Jiankang Li, Yang Chen, Yi Tang, Xiaofei Liu, Chun-Su Yuan

**Affiliations:** 1 Key Laboratory of Modern Preparation of TCM, Ministry of Education, Jiangxi University of Traditional Chinese Medicine, Nanchang, 330004 China; 2 Tang Center for Herbal Medicine Research, and Department of Anesthesia & Critical Care, The University of Chicago, Chicago, IL 60637 USA; 3 Research Center for the Resourcing of Traditional Chinese Medicine and Minority Medicine, Jiangxi University of Traditional Chinese Medicine, Nanchang, 330004 China

**Keywords:** *Aristolochiae Fructus*, Honey-fried *Aristolochiae Fructus*, Aristolochic acids, Pharmacokinetics, HPLC-MS/MS

## Abstract

**Background:**

*Aristolochiae Fructus* (AF) and honey-fried *Aristolochiae Fructus* (HAF) have been used in China for a long time as anti-tussive and expectorant drugs. Few clinical cases have been reported to be associated with the toxicity of AF and HAF, although relatively high amounts of aristolochic acids (AAs) have been found in them. Our previous experiments have verified from the chemical changes and from traditional toxicology that honey-processing can significantly reduce the toxicity of AF. To further elucidate the detoxification mechanism of honey-processing, comparative pharmacokinetics of AAs in AF and HAF are performed in this study.

**Methods:**

An HPLC-MS/MS (high-performance liquid chromatography-tandem mass spectrometry) method was developed and validated for the determination of AA I, AA II, AA C, AA D and 7-OH AA I in rat plasma. The multi-component pharmacokinetics of AAs in AF and HAF extracts were investigated after the oral administration of three doses to rats. The relative pharmacokinetic parameters were compared systematically.

**Results:**

The five AAs shared a similar nonlinear PK (pharmacokinetic) process. They involve rapid absorption and elimination, and they were fit into a two-compartmental open model. Some significant pharmacokinetic differences were observed between the AF and HAF groups: the *C*_max_ and AUC values of AA I and AA II in the AF groups were much higher than those of the HAF groups.

**Conclusions:**

Honey-frying technology can reduce the toxicity of AF by significantly decreasing the absorption of AA I and AA II. The PK parameters obtained in this work could provide valuable references for the toxicity research and clinical use of *Aristolochiaceae* herbs, including AF and HAF.

**Graphical abstract:**

Process diagram of comparative pharmacokinetics study
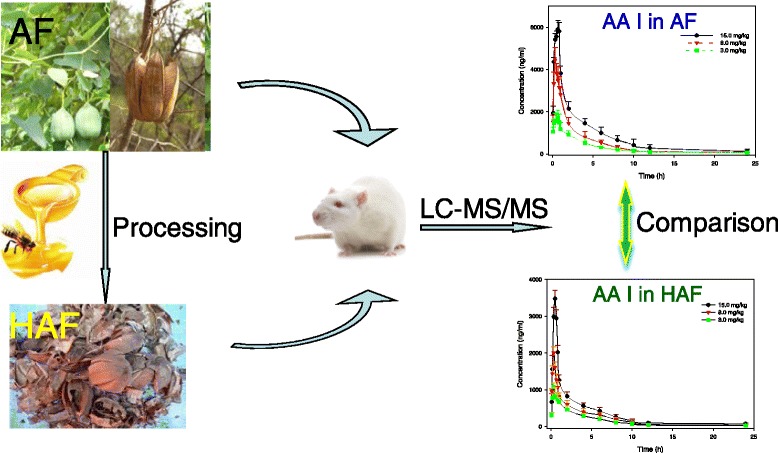

**Electronic supplementary material:**

The online version of this article (doi:10.1186/s12906-017-1626-2) contains supplementary material, which is available to authorized users.

## Background

*Aristolochiae Fructus* (AF), the dry-ripe fruit of perennial herb *Aristolochia contorta* BGE. or *Aristolochia debilis* SIEB. *et* ZUCC., has been used in China for thousands of years as an anti-tussive and expectorant drug with significant curative effects [1]. The crude AF is bitter-cold and can cause adverse reactions, such as nausea and vomiting. Honey-Fried *Aristolochiae Fructus* (HAF), the processed product of AF, has a stronger efficacy due to its ability to moisten the lungs and relieve coughs. The honey processing also improves the taste of AF, which works to prevent vomiting; there is a saying that “frying with honey makes the herbs sweet, mitigatory, and lung-moistening” [2]. Consequently, HAF is more frequently used than AF in the clinic.

Since the first aristolochic acid nephropathy (ANN) case, aristolochic acids (AAs) have been proven to be nephrotoxic [3, 4], carcinogenic [5, 6] and mutagenic [5, 7]. Therefore, most of the AAs-generating herbs and herbal preparations have been banned in many countries, including China. Recent reports [8–14] showed that the AAs contents in some *Aristolochiaceae* (*A.*) herbs are in the following order: *A. manshuriensis*>*A. fangchi*>*A. Ridix*>*A. Fructus*>*A. Herbra*. Few clinical cases were reported to be associated with the toxicity of AF and HAF, and continue to be listed in Chinese Pharmacopoeia [1]. Although relatively high amountsof AAs exist in AF, only moderate toxicity was observed during the acute and subacute toxicity tests of its extract [15].

Drug processing is a traditional pharmaceutical technology in traditional Chinese medicine, and it plays an important role in reducing the toxicity of traditional drugs. Our previous experiments have found that the honey-frying method can significantly reduce the contents of AAs in AF [16]. However, even when the contents of AAs were equivalent, the toxic effect of the HAF extract was much weaker than that of the AF extract, and the results suggested there should be another detoxification pathway [15]. To understand the phenomenon of “low toxicity” [15] and “a relatively high content of AAs” [8–14], it is necessary to investigate the pharmacokinetic or toxicokinetic characteristics of AF and HAF.

Although the nephrotoxicity and mutagenic mechanism of AAs are well studied, knowledge of the pharmacokinetic characteristics of AAs and related herbs is still limited. Nowadays, the pharmacokinetic studies about AAs focus mainly on AA I [17–19] and/or AA II [20–25]; the other AAs are seldom involved. Very recently, the preliminary pharmacokinetics of 4 AAs in AF have been investigated by the CPE -HPLC (cloud point extraction-high performance liquid chromatography) method [26]. However, the method is tedious and produces data with relatively poor sensitivity. Therefore, in order to comprehensively investigate the kinetics of multiple components in AF and HAF, a more simple, fast, sensitive and effective analytical method is necessary for the determination of AAs in biological samples.

Advances in sample pretreatment and analytical techniques have improved analysis time, sensitivity and efficiency. A number of chromatography methods have been reportedly used for the determination of AAs in herbal plants [9–15] and biological samples [26–30]. Among these, high performance liquid chromatography-tandem mass spectrometry (HPLC-MS/MS) has been employed for the quantitative analysis of AA I [19] and AA II [22, 29] in biological matrices. HPLC-MS/MS is notable for its better specificity, sensitivity and speed.

In previous experiments, we found that honey-frying technology could markedly reduce the contents of AAs in AF and reduce its toxic effects and side effects. We also discussed the detoxification mechanism of AF in terms of two aspects: chemistry [16] and traditional toxicology [15]. The primary goal of this work is to examine the detoxification mechanism of AF from the kinetic viewpoint using the honey-processing method. Firstly, an HPLC-MS/MS method was developed and well validated for the determination of five AAs (AA I, AA II, AA C, AA D and 7-OH AA I) in rat plasma. Secondly, based on the proposed analytical method, a comparative study was performed on the multi-component pharmacokinetics of AF and HAF extracts after the oral administration of three doses in rats.

## Methods

### Materials and reagents

*A. Fructus* was purchased from Haixing Chinese Herbal Pieces Ltd (Bozhou, Anhui), and was authenticated as the fruit of *Aristolochia contorta* Bunge by Professor Wuliang Yang (Jiangxi University of Traditional Chinese Medicine, JXUTCM). Voucher specimens are preserved in the Herbarium of Pharmacognosy, School of Pharmaceutical Sciences, JXUTCM. Part of AF was processed with honey to obtain HAF in our lab, according to the Chinese Pharmacopoeia [1] and the literature [16].

**Fig. 1:**
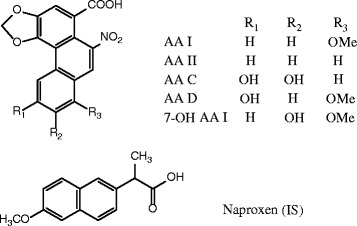
Structures of five AAs (AA I, AA II, AA C, AA D and 7-OH AA I) and naproxen (internal standard, IS)

Naproxen was purchased from the National Institute for Food and Drug Control (Beijing, China). Five AAs, shown in Fig. 1, were separated and purified in our laboratory and their purities were no lower than 98% [13, 26]. Methanol and acetonitrile were HPLC grade (Fairfield, OH, USA). Purified water (Wahaha Group, Hangzhou, China) was used throughout the experiments. All other chemical reagents were of analytical grade.

### Animals

Male Sprague–Dawley (SD) rats (Certificate No. SCXK (Hunan) 2011-001) weighting 200–220 g were purchased from Hunan Silaike Jingda Experimental Animal Ltd (Changsha, China). The animals were kept in a controlled breeding room with the following conditions: a temperature of 22 ± 2 °C, a relative humidity of (65 ± 5)%, and a 12 h light–dark cycle. The Experimental Animal Ethic Committee of JXUTCM approved all animal protocols. The animal experiments were carried out according to the European Community guidelines for the use of experimental animals.

### Preparation of stock solution, calibration standards and quality control samples

The stock solutions of the mixed standards were prepared in methanol with the concentrations of AA I (9.15 μg/mL), AA II (8.00 μg/mL), AA C (7.45 μg/mL), AA D (9.20 μg/mL) and 7-OH AA I (8.20 μg/mL). Working standards were prepared freshly by diluting the stock solution with methanol. Naproxen stock solution (1.0 mg/mL) was dissolved in methanol, and was used as an internal standard (IS). Using the stock solutions of the mixed standard and internal standard, a series of working standard solutions with gradient concentrations of the derivatives and 1.0 μg/mL of internal standard were prepared.

The relative calibration standards in plasma were prepared by spiking 20 μl of the corresponding standard solutions into 200 μl blank rat plasma. The quality control (QC) samples used in the method validation were prepared with the same procedures as the calibration standard.

### Sample preparationof the extract and rat plasma

The dried AF (800 g) and HAF (928 g) were put in a drug-decocting machine, respectively. The drugs were extracted twice, the first time with 10 times the amount of 95% ethanol for 2 h, and the second time with 8 times the amount of ethanol for 1 h. Then the two decoctions were merged and evaporated to dryness under vacuum.

An aliquot (100 μl) of rat plasma was spiked with 10.0 μL IS, and vortexed with 300 μL acetic ether for 10 s before the supernatant was transferred into centrifuge tube. The extraction was repeated with 200 μL acetic ether. The combined organic layer was evaporated to dryness under a gentle stream of nitrogen at RT. The residue was resolved in 100 μL methanol and centrifuged at 15,800 × g for 10 min. The supernatant was used for HPLC-MS/MS analysis.

### Apparatus and HPLC-MS/MS conditions

The Agilent 1290 Infinity Binary LC system consists of an autosampler, column oven, a degasser, and a binary solvent manager (Agilent Technologies, CA, USA). The Agilent 6410 triple-quadrupole mass spectrometer was equipped with an electrospray ionization (ESI) source. The HPLC-ESI-MS/MS system was controlled by MassHunter Workstation (Agilent Technologies, CA, USA).

Chromatographic separation was performed by an Acquity C_18_ column (4.6 × 100 mm,3.5 μm) (Agilent Technologies, USA). The mobile phase was composed of water (containing 10 mM ammonium formate and formic acid, pH 3.0) (solvent A) and ACN (solvent B). The gradient profile of B was: 0–10 min, 35–38%; 10–11 min, 38– 60%; 11–17 min, 60–35%B; 17–18 min, 35%. The flow rate was kept at 0.5 mL/min. The column oven was set at 30 °C and the injection volume was 10 μL.

The mass spectrometer was operated in the positive ion electrospray mode with multiple reaction monitoring (MRM). The optimal MS parameters were set at the following parameters: a desolvation gas temperature of 450 °C, desolvation gas flow of 10 L/min, a nebulizer gas of 40 psi and a capillary voltage of 4000 V. The mass spectrometry conditions of AA I, AA II, AA C, AA D, 7-OH AA I and IS are summarized in Additional file 1: Table S1.

### Method validation

This method was validated as per the current ICH guidelines. To assess the linearity ranges, a series of the mixed standard solutions (seven concentration levels) were prepared in triplicate. Each calibration curve (*y* = a*x* + b) was established by plotting the peak area ratio of analyte to IS (*y*) against the concentrations (*x*) of the calibration solution with a least square linear regression analysis. The correlation coefficient (r^2^) of the calibration curve should be >0.990 to satisfy linearity requirements. The limit of detection (LOD) and limit of quantification (LOQ) were determined when the peak height was three times and ten times the background noise, respectively.

The intra- and inter-day precisions and accuracies were calculated by an analysis of variance based on the replicate analysis of QC samples, and the work was accompanied by a standard calibration curve on each analytical run. The precision and accuracy were required to be within ±20% (relative standard deviation, RSD%) for the lower limit of quantification (LLOQ) and within ±15% (RSD%) for other concentrations. Precisions were evaluated under the optimal conditions six times within the same day for intra-day variance and six different days for inter-day variance. The accuracy was determined by calculating the percentage deviation of the observed concentrations from the spiked concentrations and expressed as a relative error (R.E.%). The recovery tests were studied by spiking the known content of the mixed standard into the blank plasma and calculated as follows: (mean measured concentration)/(spiked concentration) × 100%.

The extraction recoveries of five analytes were assessed by comparing the mean peak areas of QC samples free of IS (A) with those of the spike-after-extraction samples (B) at the same concentrations (*n* = 6). Likewise, the matrix effects were tested by comparing the peak areas of B with those of the corresponding concentration of mixed standard solution (dissolved in methanol) (C). There was no matrix effect if the ratio was between 85~115%.

The QC samples were assayed under several different conditions to evaluate the stability of AAs in rat plasma. The freeze-thaw stability of the analyte was determined over three freeze-thaw cycles. In each freeze-thaw cycle, the samples were frozen and stored at –20 °C for 24 h, then thawed at room temperature. To evaluate the long-term stability of AAs, the plasma samples were stored at –20 °C for 14 days. For the short-term stability, fresh plasma samples were kept at room temperature for 24 h before the sample preparation. After keeping the samples at room temperature for 24 h, post-preparation stability was tested. Stability was evaluated by comparing the mean concentration of the stored samples with the mean concentration of those prepared freshly. The stability data were acceptable when the bias was within ±15% of the actual value.

### Pharmacokinetic experiment

The rats were randomly divided into 6 groups (low, middle and high doses of AF and HAF; *n* = 6). After a week of acclimation, the animals were fasted for 12 h with free access to water. Then, AF and HAF extracts were administered to the rats. The extracts were suspended in 0.5% CMC–Na solution, and the oral doses (according to AA I in the extracts) were 3.0 mg/kg, 8.0 mg/kg and 15.0 mg/kg, respectively.

Blood samples were collected from the ocular vein using dried heparinized tubes at 0, 0.083, 0.17, 0.33, 0.50, 0.67, 0.83, 1, 2, 4, 6, 8, 10, 12 and 24 h from the start. The animals received fluid replacement (sterile isotonic saline) after every two samplings since the fifth collecting. The samples were then immediately centrifuged at 3100 × g for 10 min. The plasma obtained was frozen and stored at –20 °C until analysis.

All statistical analyses were performed using the SPSS 11.0 software (Chicago, USA). *P* values <0.05 were considered statistically significant. Pharmacokinetic analysis was performed using the proprietary DAS 3.0 software (Mathematical Pharmacology Professional Committee of China, Shanghai, China).

## Results and discussion

### Sample preparation method

Some sample preparation methods have been reported for the AAs in biosamples, including protein precipitation [25, 29], liquid-liquid extraction (LLE) [19, 24, 27, 28], solid-phase extraction [31, 32], liquid-phase microextraction [33] and cloud-point extraction [26]. In this work, the deproteinized methods (with methanol or acetonitrile) and the liquid–liquid extraction (with ethyl acetate and chloroform) were investigated, and the extraction recoveries and matrix effects were selected as the evaluation indexes. No significant differences were observed between the extraction recoveries of these methods. However, the matrix effect of the LLE with ethyl acetate was lower than that of protein precipitation. Ethyl acetate was consequently selected as the extraction solvent due to its lesser toxicity and good volatility. As shown in Additional file 2: Table S2, the current method was provided with suitable extraction recoveries and matrix effects.

### Selection of internal standard

In our early experiments, indomethacin was used once as an internal standard to quantify the AAs in biosamples. Gu *et al*. [29] reported the feasibility of naproxen as IS when analyzing AA I and AA II in the rat plasmas. In this work, the retention behavior and MS characteristics of indomethacin and naproxen were investigated. Indomethacin has a weaker polarity and a longer retention time than the AAs do. However, the retention time of naproxen is between that of AA D and AA I, with a suitable MS response. In order to shorten the analysis time, naproxen was selected as the internal standard substance.

### Optimum chromatography and mass spectrometry conditions

Aristolochic acids are a mixture of structurally related nitrophenanthrene carboxylic acids with a weak polarity and low ionization efficiency [12, 34]. These compounds have very similar molecular structures; 7-OH AA I and AA D are isomers and difficult to separate in a short time. To get optimum chromatography and mass spectrometry conditions, various influencing factors such as the chromatographic column, the mobile phase, and MS parameters were optimized.

To obtain a good separation, several analytical columns, including Acquity C_18_ (4.6 × 100 mm, 3.5 μm) (Agilent Technologies), YMC-Tiart C_18_ (3.0 × 150 mm, 3.0 μm) (YMC Co. LTD, Tokyo, Japan) and Welch ultimate® UHPLC XB-C_8_ (2.1 × 100 mm, 2.1 μm) (Welch Materials, Shanghai, China) were investigated. Agilent columns were found to have better separation and lower column pressure and therefore were used in subsequent experiments. Based on previous work [12, 22, 29, 34], chromatographic conditions such as the constituents and the gradient profiles of the mobile phase were further improved to adapt the separation and detection of AAs in rat plasma. In addition, the optimum mobile phase was water (containing 10 mM ammonium formate and formic acid, pH 3.0) and acetonitrile.

**Fig. 2:**
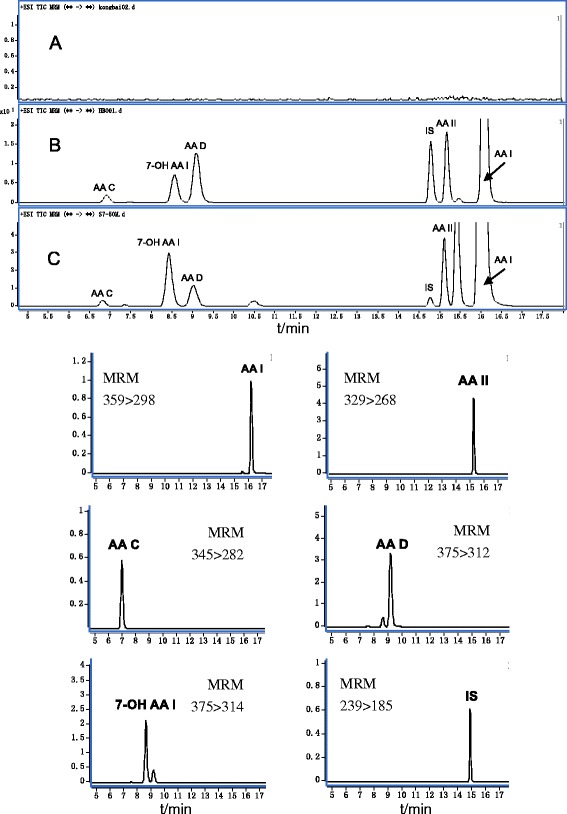
Typical MRM chromatograms of blank plasma (**a**), blank plasma spiked AAs and IS (**b**), and plasma samples at 30 min after administration (**c**)

In the experiment, the studied AAs were found to easily form ammonia adducts [M+NH_4_]^+^ (Additional file 3: Figure S1). Thus, the precursor ions were set at *m/z* 359 [AA I+NH_4_] ^+^, *m/z* 329 [AA II+ NH_4_]^+^, *m/z* 345 [AA C+NH_4_]^+^, *m/z* 375 [AA D+NH_4_]^+^ and *m/z* 375 [7-OH AA I+NH_4_]^+^ to provide the best detection sensitivity. The most intense precursor–product transitions were selected as the quantification transitions in the multiple reaction monitoring (MRM) mode. The product ions with higher abundances were selected according to the fragmentation of the mass spectra of the above precursor ions, and the fragment ions at *m/z* 298 [AA I+H-CO_2_]^+^, *m/z* 268 [AA II+H-CO_2_]^+^, *m/z* 282 [AA C+H-NO_2_]^+^, *m/z* 312 [AA D+H-NO_2_]^+^ and *m/z* 314 [7-OH AA I+H-CO_2_]^+^ were therefore selected as the product ions. It can be seen from Fig. 2, that no interference peaks in the chromatograms from endogenic metabolites of rat blood were observed. Furthermore, a good separation of AAs was obtained under optimal conditions. The results indicate the proposed method has good specificity.

### Method validation

Typical chromatograms of blank, spiked plasma and plasma samples are shown in Fig. 2. The developed method results in 6 single sharp peaks at the retention time of 6.9, 8.6, 9.2, 14.8, 15.2 and 16.2 min. No interference was found between endogenous compounds or xenobiotics and AAs.

The linearity parameters of five AAs in rat plasma are summarized in Table 1. Within the investigated linear range, good linearity data was obtained with correlation coefficients >0.998. The results indicate that the method has suitable sensitivity with LODs downward of 0.3 ng/mL and LLOQs downward of 0.73 ng/mL.

**Table 1:** Linear parameters of five AAs in rat plasma

AAs	Linear equation	*r* ^2^	Linear range (ng/mL)	LOD (ng/mL)	LLOQ (ng/mL)
AA I	*Y*_*1*_ = 0.0227*X*_*1*_-0.64	0.9998	1.46~9150	0.3	0.73
AA II	*Y*_*2*_ = 0.0081*X*_*2*_-0.1013	0.9999	3.20~8000	1.2	3.20
AA C	*Y*_*3*_ = 0.0021*X*_*3*_-0.0126	0.9986	74.5~7450	23.8	74.5
AA D	*Y*_*4*_ = 0.0094*X*_*4*_-0.0522	0.9996	14.7~9200	3.7	14.7
7-OH AA I	*Y*_*5*_ = 0.0074*X*_*5*_-0.0615	0.9997	32.8~8200	13.1	32.8

The accuracy and precision results are shown in Table 2. The analytical precisions were less than 15%, the relative errors were within −12.5~13.3%, and the recoveries were within 87.6~113.4%. These data indicate that the HPLC-MS/MS method provides suitable, reproducible, accurate, and reliable data and can be used as an analytical tool of AAs in rat plasma.

**Table 2:** Precision, accuracy and recovery of AAs in rat plasma (*n* = 6)

Compound	Concentration (ng/mL)	Precision (RSD%)		Accuracy (R.E.%)	Recovery (%)
		Intra-day	Inter-day		
AA I	1.83	0.7	1.4	12.2	112.1
	457.5	8.5	13.5	5.9	105.9
	1830	14.1	13.2	2.1	102.1
AA II	8.00	2.9	3.2	−2.5	97.6
	400	10.9	7.9	8.2	108.1
	3200	13.1	4.6	−3.7	96.4
AA C	186.3	10.9	4.7	−8.4	91.7
	372.5	10.9	13.6	4.2	104.2
	2980	6.2	7.7	−6.2	93.9
AA D	36.8	11.9	15.3	−12.5	87.6
	460	12.3	8.8	13.3	113.4
	3680	11.1	13.6	−9.2	90.8
7-OH AA I	82.0	11.3	13.3	3.0	103.0
	410	13.8	7.2	−2.8	97.2
	3280	6.2	7.3	7.7	107.7

The stability data under different experimental conditions are summarized in Table 3. The detection variabilities expressed as RSDs (%) were less than 14.3%, which suggested that rat plasma samples containing AAs were stable under routine laboratory conditions and no additional procedures were necessary to stabilize the sample for pharmacokinetic studies.

**Table 3:** Stability of AAs in rat plasma expressed as RSD% (*n* = 5)

Compound	Concentration (ng/mL)	Freeze-thaw stability (RSD/%)	Long-term stability (RSD/%)	Short-term stability (RSD/%)	Post-preparation stability (RSD/%)
AA I	1.83	1.1	0.28	0.58	0.65
	457.5	10.3	6.9	8.7	2.0
	1830	12.9	7.6	6.5	4.5
AA II	8.00	1.3	1.2	5.0	4.4
	400	10.2	8.2	5.5	3.6
	3200	14.3	7.3	9.3	6.5
AA C	186.3	5.8	8.3	14.0	9.2
	372.5	7.3	9.9	12.6	8.5
	2980	12.9	12.0	13.0	7.0
AA D	36.8	4.8	5.2	5.0	4.5
	460	8.4	2.5	11.7	3.1
	3680	14.3	12.7	8.6	2.8
7-OH AA I	82.0	6.2	2.0	7.1	5.1
	410	12.3	6.4	11.8	4.2
	3280	13.4	11.1	9.1	6.7

### Contents of AAs in extract and dose of intragastric administration

The dry extract yield of AF and HAF were 2.87 and 17.04%, respectively. The five main aristolochic acids in the extracts of AF and HAF were determined by an improved HPLC method [26], and the content results are summarized in the Additional file 4: Table S3. The results showed that the contents of AAs in the AF extract were much higher than that in the HAF extract. Even after taking into consideration the extract, the content of AAs in HAF decreased significantly compared to the content in AF, which indicated that the honey-frying processing technology reduces the content of AAs in AF [15].

In previous pharmacokinetic studies of AA I and/or AA II, the dose range was about 5~15 mg/kg [19–22, 24]. In this study, three doses were used, equivalent to AA I content in the extract: low dose (3.0 mg/kg), middle dose (8.0 mg/kg) and high dose (15.0 mg/kg). Then, the relative AF and HAF extracts were weighed out to prepare the drug solutions for the rats.

### Pharmacokinetic study of the five AAs

**Fig. 3:**
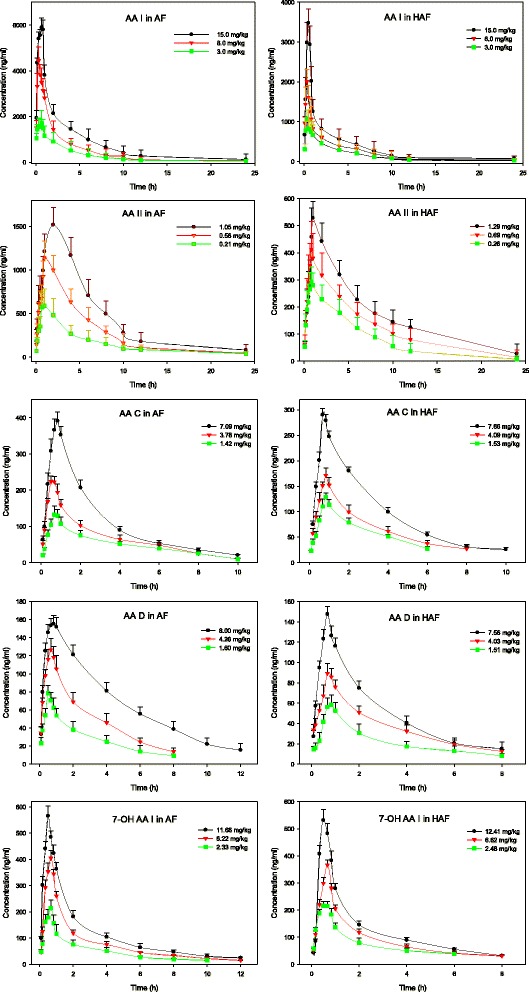
Mean plasma concentration-time profile of five AAs in rats after oral administration of AF & HAF extracts at three doses (*n* = 6)

After the oral administration of AF and HAF extracts, rat plasma samples were prepared and analyzed as described above. The typical chromatograms were shown in Fig. 2. Five main AAs can be detected in rat blood with the proposed HPLC-MS/MS method. The mean plasma concentration–time profiles of AAs after the oral administration of AF and HAF extracts are shown in Fig. 3. The mean pharmacokinetic parameters of five AAs in AF and HAF are summarized in Tables 4, 5 and Additional files 5, 6, 7: Tables S4-S6. The maximum concentration (*C*_max_) was the experimentally-observed value. The other pharmacokinetic parameters of the five analytes were fitted by DAS 3.0 software.

**Table 4:** PK parameters of AA I in rats after oral administration of AF and HAF (mean ± SD, *n* = 6)

Parameter	Unit	Low-dose		Mid-dose		High-dose	
		AF	HAF	AF	HAF	AF	HAF
Dose	mg/kg	3.0	3.0	8.0	8.0	15.0	15.0
*C*_max_	μg/L	1704.0 ± 570.8	1072.3 ± 223.1*	3823.8 ± 450.3	2002.5 ± 312.8*	5891.3 ± 425.5	3474.3 ± 352.3*
T_max_	h	0.50 ± 0.09	0.39 ± 0.08	0.56 ± 0.09	0.42 ± 0.08	0.69 ± 0.10	0.47 ± 0.08
*t*_1/2z_	h	3.50 ± 0.50	3.22 ± 0.46	5.58 ± 0.65	4.72 ± 0.52	5.46 ± 0.53	5.32 ± 0.54
AUC_(0-∞)_	μg/L · h	6080.4 ± 1263.2	5227.0 ± 895.6*	12770.7 ± 1857.5	8985.3 ± 1784.9*	26844.6 ± 3264.7	11782.2 ± 1769.2*
V_z_/F	L/kg	2.85 ± 0.15	2.75 ± 0.24	5.40 ± 0.48	4.58 ± 0.37	7.13 ± 0.57	5.53 ± 0.52
CL_z_/F	L/h/kg	0.57 ± 0.06	0.59 ± 0.08	0.65 ± 0.09	0.68 ± 0.07	0.87 ± 0.12	0.72 ± 0.08

*P < 0.05 compared with AF group

**Table 5:** PK parameters of AA II in rats after oral administration of AF and HAF (mean ± SD, *n* = 6)

Parameter	Unit	Low-dose		Mid-dose		High-dose	
		AF	HAF	AF	HAF	AF	HAF
Dose	mg/kg	0.21	0.26	0.56	0.69	1.05	1.29
*C*_max_	μg/L	590.5 ± 191.1	293.0 ± 55.2*	1137.1 ± 193.1	411.8 ± 104.2*	1518.0 ± 200.0	528.2 ± 62.4*
T_max_	h	1.03 ± 0.18	0.81 ± 0.14	1.33 ± 0.21	0.81 ± 0.16	1.83 ± 0.29	0.93 ± 0.19
*t*_1/2z_	h	4.98 ± 0.55	4.94 ± 0.61	5.06 ± 0.49	4.95 ± 0.53	5.16 ± 0.52	5.39 ± 0.48
AUC_(0-∞)_	μg/L · h	3958.9 ± 1002.8	2716.2 ± 795.2*	7824.5 ± 1975.8	3874.5 ± 1201.6*	12709.2 ± 2964.7	5628.0 ± 1693.4*
V_z_/F	L/kg	0.49 ± 0.06	0.55 ± 0.07	0.48 ± 0.05	0.49 ± 0.06	0.82 ± 0.09	0.72 ± 0.08
CL_z_/F	L/h/kg	0.07 ± 0.02	0.08 ± 0.02	0.07 ± 0.02	0.07 ± 0.02	0.11 ± 0.03	0.09 ± 0.02

*P < 0.05 compared with AF group

The five AAs shared some similar pharmacokinetic parameters, which may be due to the similarity of their structures. They were absorbed quickly and their T_max_ values were in the range of 0.43~1.83 h (see Fig. 3, Tables 4, 5 and Additional files 5, 6, 7: Tables S4-S6). Similar metabolisms were observed for the studied AAs and relative metabolites, including aristolactams and DNA adducts, were detected in the rat urine, feces and kidney tissues according to the improved analytical methods [28, 32, 35]. As can be seen from Fig. 3, they were also excreted rapidly. The physiological disposition conformed to a two-compartmental open model of the five AAs in rats fitted by the DAS 3.0 software. No good linear relationships were observed between *C*_max_ and AUC for the doses. These parameters suggest that the pharmacokinetics of AAs in rats are nonlinear.

There were also some differences between the parameters of various AAs. The ratios (AUC_(0-∞)_/dose) of AA I and AA II were higher than those of the other AAs, and the*t*_1/2z_values were in the following order from large to small: AA I, AA II, 7-OH AA I, AA D and AA C. The results show that the absorption rates of AA I and AA II were higher than those of the others, and more clearance time was needed. These differences can partially explain why AA I and AA II are the most toxic compounds of the total AAs from a pharmacokinetic viewpoint.

### Pharmacokinetics comparisons between AF and HAF

Some significant pharmacokinetic differences were observed between the AF and HAF groups (see Fig. 3, Tables 4, 5 and Additional files 5, 6, 7: Tables S4-S6). The parameters, such as *C*_max_ and AUC (including AUC _(0-t)_ and AUC_(0-∞)_), of AA I and AA II in the AF groups were are much higher than those of the HAF groups (Tables 4 and 5), which indicates that the absorption rates of AA I and AA II by rats decreased significantly in HAF groups (*p* < 0.01). These results imply that honey-frying could reduce the absorbability of AA I and AA II, thereby alleviating the toxic effects caused by herbal plants containing AAs.

The other pharmacokinetics differences are not so significant. Firstly, relatively small differences were observed for the other PK parameters of AA I and AA II between the AF and HAF groups, such as V_z_ and CL_z_. Secondly, no very significant differences were observed for the parameters of the other three AAs (AA C, AA D and 7-OH AA I) between the AF and HAF groups (Additional files 5, 6, 7: Tables S4-S6). Thirdly, the similar metabolites of the AAs are detected in the rat urine, feces and kidney tissues of AF and HAF groups, which indicate AAs in AF and HAF groups experienced a similar metabolic pathway.

In summary, honey-frying affected the physiological disposition of AAs in rats, especially the absorption of AA I and AA II. But how the physiological action was changed by honey-frying requires further investigation. Berenbaum’s group once reported that honey constituents can up-regulate detoxification and immunity genes [36], but the regulation should be a relatively long process and might not occur in the current PK experiments. According to the previous experiments [15, 16] and the current results, we can now deduce the detoxification mechanism of AF using honey-processing. It was found that the significant content decrease of AAs [16]; and the remarkable absorption decreases of AA I and AA II.

## Conclusions

Honey-frying technology can reduce the toxicity of AF by decreasing the absorption of AA I and AA II, and by reducing the content of AAs. The results have validated the scientific connotation of the honey-frying technology of AF. HAF, therefore, is recommended in clinical use instead of its crude drugs (AF) because of its increased safety. The PK parameters obtained in this work could provide valuable references for toxicity research and the clinical use of *Aristolochiaceae* herbs, including AF and HAF.

## Additional files

Additional file 1: Table S1. The mass spectrometry conditions of six compounds. (DOC 31 kb)
Additional file 2: Table S2. The recoveries and matrix effects of AAs with ethyl acetate extraction. (DOC 38 kb)
Additional file 3: Figure S1. Structures, mass spectrums and proposed main fragmentation pathways of the compounds. (DOCX 269 kb)
Additional file 4: Table S3. The contents (mg/g) of the five AAs in extract powder of AF and HAF. (DOC 30 kb)
Additional file 5: Table S4. PK parameters of AA C in rats after oral administration of AF and HAF. (DOC 38 kb)
Additional file 6: Table S5. PK parameters of AA D in rats after oral administration of AF and HAF. (DOC 36 kb)
Additional file 7: Table S6. PK parameters of 7-OH AA I in rats after oral administration of AF and HAF. (DOC 37 kb)

## References

[1] Chinese Pharmacopoeia Commission. Chinese Pharmacopoeia. 2015 edn, Beijing: China Medical Science Press; 2015.

[2] Dong LS, Shang MY, Cai SQ (2003). The study of Aristolochiae Fructus variety, production place examination and processing methods of change in history. Chin J Chin Mater Med.

[3] Vanherweghem JL, Tielemans C, Abramowicz D, Depierreux M, Vanhaelen-Fastre R, Vanhaelen M, Dratwa M, Richard C, Vandervede D, Verbeelen D, Jadoul M (1993). Rapidly progressive interstitial renal fibrosis in young women: association with slimming regimen including Chinese herbs. Lancet.

[4] Aydin S, Dekarirelle AF, Ambroise J, Durant JF, Henstersprente M, Guiot Y, Cosyns JP, Gala JL (2014). Unambiguous detection of multiple TP53 gene mutations in AAN-associated urothelial cancer in Belgium using laser capture microdissection. Plos One.

[5] Nortier J, Pozdzik A, Roumeguere T, Vanherweghem JL (2015). Aristolochic acid nephropathy (“Chinese herb nephropathy”). Nephrol Ther.

[6] Chen CH, Dickman KG, Moriya M, Zavadil J, Sidorenko VS, Edwards KL, Gnatenko DV, Wu L, Turesky RJ, Wu XR, Pu YS (2012). Aristolochic acid-associated urothelial cancer in Taiwan. Proc Natl Acad Sci U S A.

[7] Kwak DH, Lee S (2016). Aristolochic acid I causes testis toxicity by inhibiting Akt and ERK1/2 phosphorylation. Chem ResToxicol.

[8] Wei F, Cheng XL, Ma LY, Jin WT, Schaneberg BT, Khan IA (2005). Analysis of aristolochic acids and analogues in medicinal plants and their commercial products by HPLC-PAD-ESI/MS. Phytochem Anal.

[9] Huang CY, Tseng MC, Lin JH (2005). Analyzing aristolochic acids in Chinese herbal preparations using LC/MS/MS. J Food Drug Anal.

[10] Zhang CY, Wang X, Shang MY, Yu J, Xu YQ, Li ZG, Lei LC, Li XM, Cai SQ (2006). Simultaneous determination of five aristolochic acids and two aristololactams in Aristolochia plants by high-performance liquid chromatography. Biomed Chromatogr.

[11] Yuan JB, Nie LH, Zeng DY, Luo XB, Tang F, Ding L, Yao SZ (2007). Simultaneous determination of nine aristolochic acid analogues in medicinal plants and preparations by high-performance liquid chromatography. Talanta.

[12] Yuan JB, Liu Q, Wei GB, Tang F, Ding L, Yao SZ (2007). Characterization and determination of six aristolochic acids and three aristololactams in medicinal plants and their preparations by high-performance liquid chromatography-photodiode array detection/electrospray ionization mass spectrometry. Rapid Commun Mass Spectrom.

[13] Yuan JB, Liu Q, Zhu WF, Ding L, Tang F, Yao SZ (2008). Simultaneous analysis of six aristolochic acids and five aristolactams in herbal plants and their preparations by high-performance liquid chromatography–diode array detection–fluorescence detection. J Chromatogr A.

[14] Xu YQ, Li XW, Liu GX, Wang X, Shang MY, Li XM, Cai SQ (2013). Comparative study of the contents of analogues of aristolochic acid in two kinds of Aristolochiae Fructus by high-performance liquid chromatography. J Nat Med.

[15] Yuan JB, Huang Q, Ren G, Shi M, Chen L, Yang WL, Chen HF, Yang M, Yang B, Yang GH, Luo XQ (2014). Acute and subacute toxicity of the extract of Aristolochiae fructus and honey-fried Aristolochiae Fructus in rodents. Biol Pharm Bull.

[16] Li ZH, Yang B, Yang WL, Chen HF, Yang M, Yuan JB, Yan ZH, Luo XQ (2013). Effect of honey-toasting on the constituents and contents of aristolochic acid analogues in Aristolochiae Fructus. J Chin Med Mater.

[17] Tsai TH, Chou CJ, Lin LC, Tsai WJ, Chen CF (1993). Determination ofaristolochicacidin rabbit plasma by high-performance liquid chromatography with photodiode-array ultraviolet detection and itspharmacokineticsapplication. J Liq Chromatogr.

[18] Su T, Qu L, Zhang CL, Cai SQ, Li XM (2004). Studies on pharmacodynamic characteristics of aristolochic acid I in rats. Chin J Chin Mater Med.

[19] Liu YM, Lin AH, Wu ZF, Ou RM, Huang HD (2010). A liquid chromatography/tandem mass spectrometry method for determination of aristolochic acid I in rat plasma. Biomed Chromatogr.

[20] Chen SM, Fan MY, Tseng CC, Ho Y, Hsu KY (2007). Pharmacokinetics and nephrotoxicity of aristolochic acid in rabbits. Toxicon.

[21] Chen XJ, Lu Q, Fang F, Wang GJ (2008). Study of pharmacokinetics of aristolochic acid I and II in rats. Chin J Chin Mater Med.

[22] Kuo CH, Lee CW, Lin SC, Tsai IL, Lee SS, Tseng YJ, Kang JJ, Peng FC, Chu L (2010). Rapid determination of aristolochic acids I and II in herbal products and biological samples by ultra-high-pressure liquid chromatography-tandem mass spectrometry. Talanta.

[23] Tian BP, Zhang LT, Yuan ZF, Liu WN, Liu HJ (2005). Pharmacokinetics of aristolochic acid A in Radix Aristolochiae and Guangxinsuhe capsules. Chin Tradt Herbal Drugs.

[24] Dolma P, Yang DH, Yu J, Wang X, Cai SQ (2009). Pharmacokinetic studies of aristolochic acid AA I and AA II in rats after intragastrical administration of Radix Aristolochiae and Muskone. J Chin Pharm Sci.

[25] Yang HY, Zheng XH, Du Y, Chen Z, Zhu DY, Lou YJ (2011). Kinetics of aristolochic acid I after oral administration of Radix Aristolochiae or Guanxinsuhe preparation in canines. J Ethnopharm.

[26] Ren G, Huang Q, Wu JG, Yuan JB, Yang GH, Yan ZH, Yao SZ (2014). Cloud point extraction-HPLC method for the determination and pharmacokinetic study of aristolochic acids in rat plasma after oral administration of *Aristolochiae Fructus*. J Chromatogr B.

[27] Yue H, Chan W, Guo L, Cai ZW (2009). Determination of aristolochic acid I in rat urine and plasma by high-performance liquid chromatography with fluorescence detection. J Chromatogr B.

[28] Yuan JB, Luo XZ, Guo ML, Wu JG, Yang WL, Yu RY, Yao SZ (2009). Determination of aristolochic acid I and its metabolites in cell culture with a hyphenated high-performance liquid chromatographic technique for cell toxicology. Talanta.

[29] Gu JP, Zhang PJ, Zhang FF, Shen HQ, Yang BC, Liang XM, Chu XG (2013). Determination of aristolochic acids in rat serum by high performance liquid chromatography-Q-TOF tandem mass spectrometry. Anal Met.

[30] Vaclavik L, Krynitsky AJ, Rader JI (2014). Quantification of aristolochic acids I and II in herbal dietary supplements by ultra-high-performance liquid chromatography-multistage fragmentation mass spectrometry. Food Addit Contam Part A Chem Anal Control Expo Risk Assess.

[31] Wei J, Guo ZM, Zhang PJ, Zhang FF, Yang BC, Liang XM (2012). A new reversed-phase/strong anion-exchange mixed-mode stationary based on polar-copolymerized approach and its application in the enrichment of aristolochic acids. J Chromatogr A.

[32] Leung EM, Chan W (2014). Noninvasive measurement of aristolochic acid-DNA adducts in urine samples from aristolochic acid-treated rats by liquid chromatography coupled tandem mass spectrometry: Evidence for DNA repair by nucleotide-excision repair mechanisms. Mutat Res.

[33] Song RJ, Pu FP, Zhou J, Sun JB, Zeng P, Zhang Q (2014). Three-phase hollow fiber liquid-phase microextraction based on a magnetofluid for the analysis of aristolochic acids in plasma by high-performance liquid chromatography. J Sep Sci.

[34] Kite GC, Yule MA, Leon C, Simmonds MSJ (2002). Detecting aristolochic acids in herbal remedies by liquid chromatography/serial mass spectrometry. Rapid Commun Mass Spectrom.

[35] Chan W, Zheng YF, Cai ZW (2007). Liquid chromatography—tandem mass spectrometry analysis of the DNA adducts of aristolochic acids. J Am Soc Mass Spectrom.

[36] Mao WF, Schuler MA, Berenbaum MR (2013). Honey constituents up-regulate detoxification and immunity genes in the western honey bee Apis mellifera. Proc Natl Acad Sci U S A.

