# Supplementary material for: Comparative studies on the multi-component pharmacokinetics of Aristolochiae Fructus and honey-fried Aristolochiae Fructus extracts after oral administration in rats
Source: BMC Complement Altern Med. 2017 Feb 10;17:107. doi: 10.1186/s12906-017-1626-2 (PMC5303205; doi:10.1186/s12906-017-1626-2)
Supplement: Additional file 1: Table S1. — The mass spectrometry conditions of six compounds. (DOC 31 kb) [file 12906_2017_1626_MOESM1_ESM.doc]

**Table** **S1** The mass spectrometry conditions of six compounds

| Compound | Presusor ion（m/z） | Product ion (m/z) | Fragmentor（V） | Collision energy (V) |
| --- | --- | --- | --- | --- |
| AA I | 359.1 | 298.1 | 95 | 6 |
| AA II | 329.0 | 268.0 | 55 | 5 |
| AA C | 345.0 | 282.0 | 85 | 5 |
| AA D | 375.1 | 312.0 | 95 | 7 |
| 7-OH AA I | 375.0 | 314.0 | 95 | 5 |
| IS | 231.2 | 185.2 | 90 | 8 |
