# Supplementary material for: Comparative studies on the multi-component pharmacokinetics of Aristolochiae Fructus and honey-fried Aristolochiae Fructus extracts after oral administration in rats
Source: BMC Complement Altern Med. 2017 Feb 10;17:107. doi: 10.1186/s12906-017-1626-2 (PMC5303205; doi:10.1186/s12906-017-1626-2)
Supplement: Additional file 2: Table S2. — The recoveries and matrix effects of AAs with ethyl acetate extraction. (DOC 38 kb) [file 12906_2017_1626_MOESM2_ESM.doc]

**Table S2** The recoveries and matrix effects of AAs with ethyl acetate extraction

| AA s | Concentration  (ng/mL) | Extraction recovery (%)  (mean±SD) | Matrix effect (%)  (mean±SD) |
| --- | --- | --- | --- |
| AA I | 4.58 | 84.0±1.9 | 79.9±2.0 |
|  | 22.8 | 83.0±2.0 | 84.9±1.6 |
|  | 36.6 | 83.3±1.8 | 90.4±1.7 |
| AA II | 4.0 | 71.7±1.8 | 76.7±2.1 |
|  | 20.0 | 83.9±1.7 | 82.2±1.9 |
|  | 32.0 | 80.7±2.0 | 86.5±1.9 |
| AA C | 3.73 | 65.4±2.1 | 89.4±2.1 |
|  | 18.6 | 77.6±2.3 | 91.8±2.1 |
|  | 29.8 | 77.4±1.9 | 87.2±2.0 |
| AA D | 4.60 | 83.6±2.2 | 89.6±2.0 |
|  | 23.0 | 84.9±1.9 | 92.5±2.1 |
|  | 36.8 | 86.1±2.0 | 87.3±1.5 |
| 7-OH AA I | 4.10 | 93.8±2.0 | 110.5±2.3 |
|  | 20.5 | 92.2±1.8 | 107.0±2.5 |
|  | 32.8 | 84.8±1.7 | 99.2±2.1 |
