# Supplementary material for: Comparative studies on the multi-component pharmacokinetics of Aristolochiae Fructus and honey-fried Aristolochiae Fructus extracts after oral administration in rats
Source: BMC Complement Altern Med. 2017 Feb 10;17:107. doi: 10.1186/s12906-017-1626-2 (PMC5303205; doi:10.1186/s12906-017-1626-2)
Supplement: Additional file 3: Figure S1. — Structures, mass spectrums and proposed main fragmentation pathways of the compounds. (DOCX 269 kb) [file 12906_2017_1626_MOESM3_ESM.docx]

5

x10

0

0.25

0.5

0.75

1

1.25

1.5

1.75

2

2.25

2.5

2.75

3

3.25

+ESI

TIC (0.136-0.188 min, 8 scans)

Frag=135.0V AA I-SCAN-135-X014.d

298.1000

359.1000

324.1000

340.3000

149.0000

114.0000

Counts vs. Mass-to-charge (m/z)

100

120

140

160

180

200

220

240

260

280

300

320

340

360

380

[M-H_2_O+H]^+^

[M+NH_4_]^+^

[M-NO_2_+H]^+^

5

x10

0

0.2

0.4

0.6

0.8

1

1.2

1.4

1.6

1.8

2

2.2

+ESI

TIC (0.141-0.227 min, 12 scans)

Frag=135.0V AA II-SCAN-135-X032.d

294.0000

268.0000

329.0000

238.1000

312.2000

149.0000

221.0000

Counts vs. Mass-to-charge (m/z)

100

120

140

160

180

200

220

240

260

280

300

320

340

360

380

[M-H_2_O+H]^+^

[M-NO_2_+H]^+^

[M+NH_4_]^+^

(Continued)

4

x10

0

0.1

0.2

0.3

0.4

0.5

0.6

0.7

0.8

0.9

1

1.1

1.2

+ESI

TIC (0.103-0.133 min, 5 scans)

Frag=135.0V AA C2-Scan002.d

282.0000

345.0000

226.1000

Counts vs. Mass-to-charge (m/z)

180

190

200

210

220

230

240

250

260

270

280

290

300

310

320

330

340

350

360

312.1000

[M-CO_2_+H]^+^

[M –NO_2_+H]^+^

[M+NH_4_]^+^

[M+NH_4_]^+^

5

x10

0

0.1

0.2

0.3

0.4

0.5

0.6

0.7

0.8

0.9

1

1.1

1.2

1.3

1.4

1.5

+ESI

TIC (0.173-0.211 min, 6 scans)

Frag=95.0V AA D-SCAN-95-X0028.d

312.0000

375.1000

279.1000

228.1000

130.1000

Counts vs. Mass-to-charge (m/z)

100

120

140

160

180

200

220

240

260

280

300

320

340

360

380

400

[M-NO_2_+H]^+^

[M-H_2_O+H]^+^

(Continued)

5

x10

0

0.2

0.4

0.6

0.8

1

1.2

1.4

1.6

1.8

2

+ESI

TIC (0.173-0.213 min, 6 scans)

Frag=135.0V 7-OH AA I-SCAN-135-X0065.d

…

375.0000

314.0000

340.0000

209.0000

149.0000

Counts vs. Mass-to-charge (m/z)

120

140

160

180

200

220

240

260

280

300

320

340

360

380

400

420

[M-H_2_O+H]^+^

[M-CO_2_+H]^+^

[M+NH_4_]^+^

[M+NH_4_]^+^

[M+H]^+^

4

x10

0

0.5

1

1.5

2

2.5

3

3.5

4

+ESI

TIC (14.618-14.827 min, 26 scans)

Frag=100.0V IS0008.d

231.2000

253.2000

275.2000

185.2000

172.1000

Counts vs. Mass-to-charge (m/z)

100

110

120

130

140

150

160

170

180

190

200

210

220

230

240

250

260

270

280

290

300

[M+H-CO-H_2_O]^+^

**Fig. S1. Structures, mass spectrums and proposed main fragmentation pathways of the compounds.**
