# Supplementary material for: Comparative studies on the multi-component pharmacokinetics of Aristolochiae Fructus and honey-fried Aristolochiae Fructus extracts after oral administration in rats
Source: BMC Complement Altern Med. 2017 Feb 10;17:107. doi: 10.1186/s12906-017-1626-2 (PMC5303205; doi:10.1186/s12906-017-1626-2)
Supplement: Additional file 4: Table S3. — The contents (mg/g) of the five AAs in extract powder of AF and HAF. (DOC 30 kb) [file 12906_2017_1626_MOESM4_ESM.doc]

**Table S3** The contents (mg/g) of the five AAs in extract powder of AF and HAF

| Sample | AA I | AA II | AA C | AA D | 7-OH AA I | Total AAs |
| --- | --- | --- | --- | --- | --- | --- |
| AF | 8.54 | 0.596 | 4.036 | 4.552 | 6.639 | 24.36 |
| HAF | 0.591 | 0.051 | 0.302 | 0.298 | 0.489 | 1.73 |
