# Supplementary material for: Comparative studies on the multi-component pharmacokinetics of Aristolochiae Fructus and honey-fried Aristolochiae Fructus extracts after oral administration in rats
Source: BMC Complement Altern Med. 2017 Feb 10;17:107. doi: 10.1186/s12906-017-1626-2 (PMC5303205; doi:10.1186/s12906-017-1626-2)
Supplement: Additional file 5: Table S4. — PK parameters of AA C in rats after oral administration of AF and HAF. (DOC 38 kb) [file 12906_2017_1626_MOESM5_ESM.doc]

**Table S4** PK parameters of AA C in rats after oral administration of AF and HAF

| Parameter | Unit | Low-dose^a^ | | Mid-dose^b^ | | High-dose^c^ | |
| --- | --- | --- | --- | --- | --- | --- | --- |
|  |  | AF | HAF | AF | HAF | AF | HAF |
| Dose | mg/kg | 1.42 | 1.53 | 3.78 | 4.09 | 7.09 | 7.66 |
| *C*_max_ | μg/L | 132.2 | 128.8 | 223.1 | 171.0 | 391.0 | 290.2 |
| T_max_ | h | 0.81 | 0.83 | 0.64 | 0.78 | 0.78 | 0.77 |
| *t*_1/2z_ | h | 2.31 | 2.32 | 2.38 | 2.21 | 2.92 | 2.72 |
| AUC_(0-∞)_ | μg/L·h | 516.2 | 388.4 | 708.6 | 530.2 | 1096.2 | 816.1 |
| V_z_/F | L/kg | 12.98 | 12.66 | 19.60 | 18.79 | 28.85 | 22.45 |
| CL_z_/F | L/h/kg | 3.85 | 3.76 | 5.70 | 5.89 | 6.85 | 5.69 |
