# Supplementary material for: Comparative studies on the multi-component pharmacokinetics of Aristolochiae Fructus and honey-fried Aristolochiae Fructus extracts after oral administration in rats
Source: BMC Complement Altern Med. 2017 Feb 10;17:107. doi: 10.1186/s12906-017-1626-2 (PMC5303205; doi:10.1186/s12906-017-1626-2)
Supplement: Additional file 6: Table S5. — PK parameters of AA D in rats after oral administration of AF and HAF. (DOC 36 kb) [file 12906_2017_1626_MOESM6_ESM.doc]

**Table S5** PK parameters of AA D in rats after oral administration of AF and HAF

| Parameter | Unit | Low-dose | | Mid-dose | | High-dose | |
| --- | --- | --- | --- | --- | --- | --- | --- |
|  |  | AF | HAF | AF | HAF | AF | HAF |
| Dose | mg/kg | 1.60 | 1.51 | 4.26 | 4.03 | 8.00 | 7.56 |
| *C*_max_ | μg/L | 78.5 | 58.6 | 126.5 | 89.2 | 155.1 | 147.5 |
| T_max_ | h | 0.56 | 0.78 | 0.56 | 0.75 | 0.64 | 0.63 |
| *t*_1/2z_ | h | 2.45 | 2.05 | 2.43 | 2.30 | 2.89 | 2.4 |
| AUC_(0-∞)_ | μg/L·h | 194.5 | 178.6 | 402.4 | 323.2 | 960.3 | 589.8 |
| V_z_/F | L/kg | 35.70 | 30.57 | 75.69 | 37.78 | 152.97 | 47.85 |
| CL_z_/F | L/h/kg | 10.11 | 9.46 | 21.56 | 11.41 | 33.62 | 12.36 |
