# Supplementary material for: Comparative studies on the multi-component pharmacokinetics of Aristolochiae Fructus and honey-fried Aristolochiae Fructus extracts after oral administration in rats
Source: BMC Complement Altern Med. 2017 Feb 10;17:107. doi: 10.1186/s12906-017-1626-2 (PMC5303205; doi:10.1186/s12906-017-1626-2)
Supplement: Additional file 7: Table S6. — PK parameters of 7-OH AA I in rats after oral administration of AF and HAF. (DOC 37 kb) [file 12906_2017_1626_MOESM7_ESM.doc]

**Table S6** PK parameters of 7-OH AA I in rats after oral administration of AF and HAF

| Parameter | Unit | Low-dose | | Mid-dose | | High-dose | |
| --- | --- | --- | --- | --- | --- | --- | --- |
|  |  | AF | HAF | AF | HAF | AF | HAF |
| Dose | mg·kg^-1^ | 2.33 | 2.48 | 6.22 | 6.62 | 11.66 | 12.41 |
| *C*_max_ | μg/L | 214.2 | 216.5 | 404.2 | 366.1 | 565.7 | 531.4 |
| T_max_ | h | 0.64 | 0.50 | 0.72 | 0.67 | 0.5 | 0.53 |
| *t*_1/2z_ | h | 3.84 | 2.75 | 3.25 | 2.55 | 3.45 | 2.94 |
| AUC_(0-∞)_ | μg/L·h | 601.9 | 620.2 | 1162.7 | 990.8 | 1321.7 | 1131.2 |
| V_z_/F | L/kg | 24.72 | 17.47 | 26.15 | 25.41 | 52.29 | 47.43 |
| CL_z_/F | L/h/kg | 4.29 | 4.88 | 5.55 | 6.55 | 10.44 | 12.44 |
